# Attenuated expression of tenascin-c in ovalbumin-challenged STAT4-/- mice

**DOI:** 10.1186/1465-9921-12-2

**Published:** 2011-01-04

**Authors:** Anna Meuronen, Piia Karisola, Marina Leino, Terhi Savinko, Kristiina Sirola, Marja-Leena Majuri, Päivi Piirilä, Ismo Virtanen, Mika Mäkelä, Annika Laitinen, Lauri A Laitinen, Harri Alenius

**Affiliations:** 1 Institute of Biomedicine/Anatomy, University of Helsinki, Biomedicum, Helsinki, Finland; 2 Finnish Institute of Occupational Health, Helsinki, Finland; 3 Laboratory for Clinical Physiology, Helsinki University Central Hospital, Finland; 4 Division of Allergy, Helsinki University Central Hospital, Finland; 5 Department of Medicine, Helsinki University Central Hospital, Finland

## Abstract

**Background:**

Asthma leads to structural changes in the airways, including the modification of extracellular matrix proteins such as tenascin-C. The role of tenascin-C is unclear, but it might act as an early initiator of airway wall remodelling, as its expression is increased in the mouse and human airways during allergic inflammation. In this study, we examined whether Th1 or Th2 cells are important regulators of tenascin-C in experimental allergic asthma utilizing mice with impaired Th1 (STAT4-/-) or Th2 (STAT6-/-) immunity.

**Methods:**

Balb/c wildtype (WT), STAT4-/- and STAT6-/- mice were sensitized with intraperitoneally injected ovalbumin (OVA) followed by OVA or PBS airway challenge. Airway hyperreactivity (AHR) was measured and samples were collected. Real time PCR and immunohistochemistry were used to study cytokines and differences in the expression of tenascin-C. Tenascin-C expression was measured in human fibroblasts after treatment with TNF-α and IFN-γ *in vitro*.

**Results:**

OVA-challenged WT mice showed allergic inflammation and AHR in the airways along with increased expression of TNF-α, IFN-γ, IL-4 and tenascin-C in the lungs. OVA-challenged STAT4-/- mice exhibited elevated AHR and pulmonary eosinophilia. The mRNA expression of TNF-α and IFN-γ was low, but the expression of IL-4 was significantly elevated in these mice. OVA-challenged STAT6-/- mice had neither AHR nor pulmonary eosinophilia, but had increased expression of mRNA for TNF-α, IFN-γ and IL-4. The expression of tenascin-C in the lungs of OVA-challenged STAT4-/- mice was weaker than in those of OVA-challenged WT and STAT6-/- mice suggesting that TNF-α and IFN-γ may regulate tenascin-C expression *in vivo*. The stimulation of human fibroblasts with TNF-α and IFN-γ induced the expression of tenascin-C confirming our *in vivo *findings.

**Conclusions:**

Expression of tenascin-C is significantly attenuated in the airways of STAT4-/- mice, which may be due to the impaired secretion of TNF-α and IFN-γ in these mice.

## Background

Allergic asthma is an inflammatory disorder of the airways characterised by episodes of airway obstruction and wheezing. Th2 cells secreting IL-4, IL-5 and IL-13 have been identified in the airways of asthmatic patients. In humans, Th2 cytokines produced in the respiratory tract lead to airway eosinophilia, high levels of serum IgE, and mast cell activation [1], which are believed to contribute to pathologies such as airway hyperresponsiveness (AHR), epithelial damage and mucus secretion. Accumulating evidence suggests, however, that airway inflammation alone may not explain the irreversible progression of the disease in some patients despite anti-inflammatory therapy [2]. Therefore, in recent years, greater effort has focused on remodelling of the airways, which entails thickening of the airway wall, depositing extracellular matrix (ECM) proteins, and altering their epithelial composition [1].

The expression of tenascin-C is increased in the airway wall in asthma. Tenascin-C belongs to the matricellular proteins and is widely expressed during embryonic development, but is generally lacking in adult tissues. Tenascin-C contributes to cell adhesion, migration and proliferation [3], participates in lung development and remodelling [3,4], and is necessary for branching morphogenesis of the bronchial tree [4,5]. Tenascin-C is down-regulated after alveolarization [5-7], and its expression is sparse or absent under the bronchial epithelium in healthy adults [8]. We have previously demonstrated that human patients with asthma show elevated levels of tenascin-C in the BM of the bronchi quite early after diagnosis [8,9]. However, very little is known about the regulation of tenascin-C or its role in asthma *in vivo*.

STAT6 and STAT4 are members of the signal transducer and activator of transcription (STAT) family of transcription factors. STAT6 is critical in mediating the effects of IL-4 and IL-13 [10,11]. Thus, STAT6 is important in Th2 lymphocyte differentiation, allergic responses such as AHR, and combating parasitic infections in mice. STAT6 induces the expression of IL-4 and down-regulates the production of IFN-γ [12]. STAT6 has been suggested as a possible new target for asthma treatment [13,14]. STAT4, on the other hand, mediates the effects of IL-12 on gene transcription, thereby promoting Th1 lymphocyte differentiation, and IFN-γ is one of its main targets [12,15]. Interestingly, STAT4 seems to also play an important role in Th2 responses [16,17].

We investigated whether one of these two distinct parts of adaptive immunity, Th1 or Th2, might play an independent role in the regulation of tenascin-C expression in experimental allergic asthma in the lung. We explored the contribution of impairment in Th1 and Th2 immunity to tenascin-C expression using STAT4-/- and STAT6 -/- mice. Furthermore, we examined the expression of Th1 (IFN-γ) cytokines, Th2 (IL-4) cytokines and TNF-α, which are all important in asthma [1] and are possible regulators of tenascin-C expression [9,18-21]. The results were tested and confirmed with human cells *in vitro*.

## Methods

### Animals

BALB/c female mice, age 6 to10 weeks and free of specific pathogens, were obtained from M&B, Denmark. STAT4-/- and STAT6-/- mice, both of Balb/c background, were purchased from Jackson Laboratory (Bar Harbor, Maine, USA). All the mice were housed under specific pathogen-free conditions and maintained on an ovalbumin-free diet at the Finnish Occupational Health Institute. All the experiments were approved by the State Provincial Office of Southern Finland.

### Sensitisation and airway challenge

On days 0 and 14, all mice were sensitised with an intraperitoneal (i.p) injection of 50 μg of ovalbumin (OVA) (Grade V; Sigma, St. Louis, MO, USA) emulsified in 2 mg of aluminium hydroxide in a total volume of 100 μl. The mice were intranasally challenged with 50 μl of PBS (controls) or with 50 μg of OVA diluted in 50 μl of PBS for three days (days 28, 29 and 30) under isoflurane anaesthesia with spontaneous breathing. Eight mice were assigned to each group.

### Determination of airway responsiveness

Airway responsiveness was assessed 24 hours after the last OVA airway challenge. A single-chamber whole-body plethysmograph system obtained from Buxco Technologies (Troy, NY, USA) was used as described [22]. Briefly, mice were placed unrestrained into a chamber and exposed for five min to nebulised PBS and subsequently to increasing concentrations of methacholine (MCh) (Sigma-Aldrich, UK) in PBS using an AeroSonic 5000 D ultrasonic nebuliser (DeVilbiss, Somerset, PA). Recordings were taken after each nebulisation for five min. The enhanced pause (Penh) values for each five-min sequence were evaluated and expressed for each MCh concentration. Baseline Penh values did not differ significantly between groups.

### Sample collections and lung preparations

The mice were killed by isoflurane overdose and the blood was drained from the hepatic vein. The chest cavity was opened, and the lungs were lavaged with 800 μl of PBS via the tracheal tube. The bronchoalveolar lavage (BAL) sample was cytospun on a slide, the cells were stained with MayGrünwald-Giemsa (MGG) stain, and the cell differentials were counted under a light microscope. The left lung was removed, quick-frozen and stored at -70°C for RNA isolation. For the immunohistological examination, half of the right lung was embedded in TissueTek OCT Compound (Sakura Finetek Europe B.V., The Netherlands), quick-frozen and stored at -70°C. The other half was fixed in paraformaldehyde and embedded in paraffin.

### Real-time quantitative RT-PCR assay

Total RNA from the lungs and cells was extracted using Eurozol Reagent (EuroClone, Italy) according to the manufacturer's instructions. Total RNA was quantified with spectrophotometry (Nanodrop, Nanodrop Technologies, Wilmington, DE, USA). The RNA was reverse-transcribed into cDNA with a High Capacity cDNA Reverse Transcription Kit (Applied Biosytems, Foster City, CA, USA). The real-time quantitative polymerase chain reaction (PCR) was performed with an Applied Biosystems 7500 Fast Real-Time PCR System, according to the manufacturer's instructions. PCR primers and probes were obtained as predeveloped assay reagents (cytokines and 18 S rRNA) or were generated (tenascin-C) with PrimerExpress version 1.5 software and ordered from Applied Biosystems. The primer and probe sequences for tenascin-C were as follows: forward primer 5'-ACC ATG CTG AGA TAG ATG TTC CAA A-3', reverse primer 5'-CTT GAC AGC AGA AAC ACC AAT CC-3', and probe 5'-ACC ACA CTC ACA GGT CTA AGG CCC GG-3'. The accumulation of PCR products was detected directly by monitoring the increase in fluorescence of the reporter dye. The signals were standardised to the internal passive reference ROX to exclude non-PCR-related fluctuations in the fluorescence. In addition, FAM signals were standardised to the endogenous reference rRNA 18S to normalise the quantification of mRNA targets for differences in total RNA.

### Immunohistochemistry

Quick-frozen samples were used for immunofluoresence. Monoclonal rat anti-mouse antibody clone MTn-12 from Sigma-Aldrich (1:200) served to detect tenascin-C and fluorescein (FITC)-conjugated anti-rat IgG (Jackson ImmunoResearch Laboratories Europe, UK) (1:100) served as a secondary antibody. The intensity of immunoreactivity for tenascin-C was graded in random order slide by slide. All the groups were graded at the same time. This resulted in grades from 0-4 (0 = no reactivity, 1 = weak reactivity, 2 = intermediate reactivity, 3 = strong reactivity, 4 = very strong reactivity). All samples were coded, and the codes were broken after completion of all the tests.

### Cells

MRC-9 cells were obtained from ATCC (Manassas, VA, USA). The cells were cultured in MEM supplemented with 10% FBS, 2 mM glutamine, 0.1 mM nonessential amino acids, 1.5 g/L of sodium pyruvate and antibiotics (Gibco, Invitrogen, Life Technologies, Carlsbad, CA, USA) at 37°C in 5% CO_2_. Cells were stimulated with TNF-α (10 ng/ml) (Biosource International, Camarillo, CA, USA) at 37°C for three and nine hours.

Primary human dermal fibroblasts isolated from adult skin were purchased from Gibco and cultured in Medium 106 (Gibco) supplemented with LSGS (Low serum growth supplement) and antibiotics (Gibco) at 37°C in 5% CO_2_. Cells were stimulated with TNF-α (20 ng/ml), IFN-γ (500 IU/ml, Immuno Tools, Friesoythe, Germany) and with a combination of TNF-α and IFN-γ for 2, 6 and 18 hours.

### Western blotting

Tenascin-C protein was analyzed by Western blot from whole-cell extracts and concentrated cell supernatants. Human primary fibroblasts stimulated with the combination of TNF-α and IFN-γ for 18 h were lysed in protein lysis buffer containing 10 mM Tris (pH7.4), 150 mM NaCl, and 25% ethylene glycol supplemented with complete mini protease inhibitor mixture (Roche Diagnostic, Indianapolis, IN). The cell extract was homogenized with ultrasound sonicator (Sanyo Electronics, San Diego, CA). Total protein concentrations were determined with Bio-Rad Dc Protein Assay (Bio-Rad Laboratories, Hercules, CA) according to the manufacturer's instructions. 20 μg of protein from lysed cell extracts and 7,5 μl of concentrated cell culture supernatants were separated with SDS-PAGE on 12% gels and transferred onto Immobilon-P Transfer Membranes (Millipore). Membranes were blocked with 5% non-fat milk in PBS and exposed to monoclonal mouse antihuman antibody 100EB2 recognizing tenascin-C [23]. After this, membrane was incubated with HRP-conjugated polyclonal goat anti-mouse immunoglobulins (Dako Cytomation). Proteins were visualized by Luminescent Image Analyzer (Image Quant LAS4000mini, GE Healthcare, Sweden). Total protein in the membrane was stained with SYPRO Ruby Protein Blot Stain (Bio-Rad Laboratories).

### Statistical Analysis

The data were analysed with GraphPadPrism Software (GraphPad Software, Inc., CA, USA). Single-group comparisons were performed with the Mann-Whitney U-test or Student's t-test when appropriate. Values for measurements are expressed as the mean ± the standard error of the mean (SEM). P values < 0.05 were considered significant.

## Results

### Tenascin-C levels are significantly elevated in the airways of ovalbumin-sensitised and challenged mice

**Figure 1:**
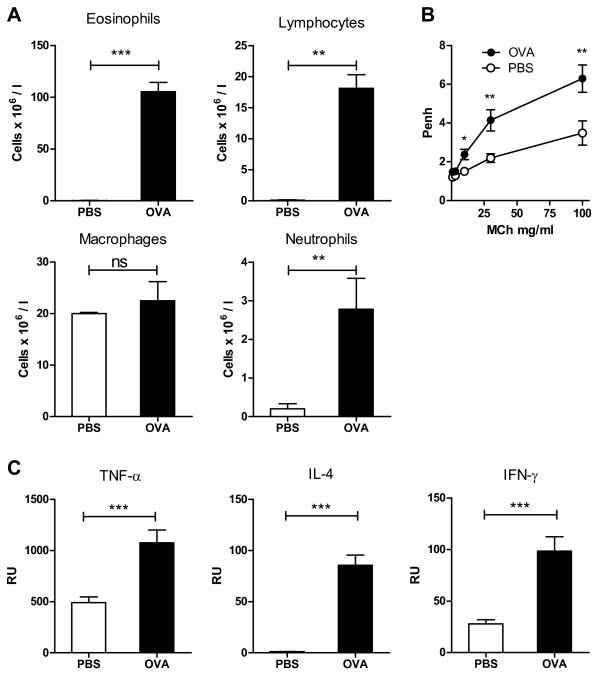
**BAL (bronchoalveolar lavage) fluid cell counts, airway hyperreactivity and the mRNA expression of cytokines in ovalbumin (OVA)-challenged wildtype Balb/c mice compared to PBS-challenged controls**. A. Absolute number of eosinophils, lymphocytes, macrophages and neutrophils in BAL fluid. B. Bronchial hyperreactivity expressed as enhanced-pause values (Penh) for each concentration of methacholine. C. Relative mRNA expression of pro-inflammatory cytokines TNF-α, Th2 cytokine IL-4 and Th1 cytokine IFN-γ compared to controls. RU = relative expression unit. Results are expressed as mean ± SEM. * p < 0.05, **p < 0.01, ***p < 0.001. n = 8 mice/group.

A significant and prominent influx of eosinophils, lymphocytes and neutrophils in BAL was observable in OVA-challenged WT mice, whereas no eosinophils and only a few lymphocytes and neutrophils appeared in the BAL of the control mice (Figure 1A). Bronchial reactivity to inhaled methacholine was significantly higher in the OVA-challenged mice than in the controls (Figure 1B). The mRNA levels of proinflammatory cytokine TNF-α, Th2 cytokine IL-4 and Th1 cytokine IFN-γ were significantly higher in the lung tissue of the OVA-challenged WT mice (Figure 1C). All these results indicate that our mouse model presents an acute allergic asthmatic response in the airways.

**Figure 2:**
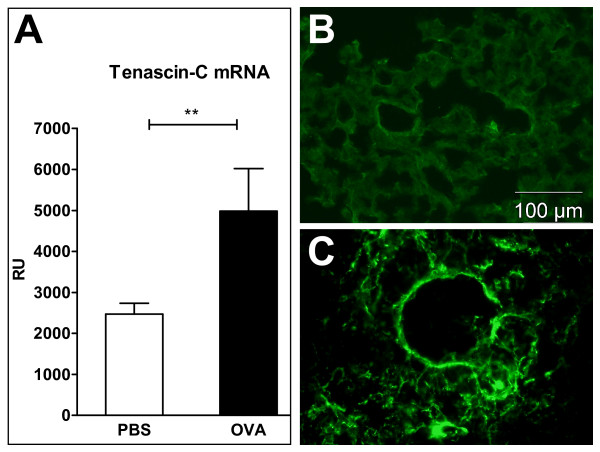
**Tenascin-C expression in wildtype mice**. A. The relative mRNA expression of tenascin-C in the lung of ovalbumin-challenged and control mice. B-C. Immunofluoresence. The immunoreactivity of tenascin-C in the lungs of control (B) and ovalbumin-challenged mice (C). The lung parenchyma was stained brightly with tenascin-C in the ovalbumin-challenged mice, but not in the control mice. All the mice showed sparse and faint immunoreactivity to tenascin-C in the veins and bronchial smooth muscle. The bronchial basement membrane tested negative for tenascin-C in both groups. RU = relative expression unit. Results are expressed mean ± SEM. ** p < 0.01, *** p < 0.001. n = 8 mice/group.

Tenascin-C mRNA levels were significantly higher in the lung tissue of the OVA-challenged WT mice than in that of the PBS-challenged mice (Figure 2A). Tenascin-C immunoreactivity was weak and sparse in the control mice, although some staining was occasionally detected in the bronchial veins, the basal layer of the epithelium, and the smooth muscle beneath the bronchial epithelium (Figure 2B). In the OVA-challenged WT mice, immunoreactivity for tenascin-C was strongly up-regulated in the alveolar structures and was evident in small spots throughout the parenchyma (Figure 2C). The larger veins were extremely immunoreactive in places in the WT mice (not shown), whereas immunoreactivity for tenascin-C in the bronchial BM was negative in all groups.

### Tenascin-C levels are significantly reduced in OVA-challenged STAT4-/- mice but normal in STAT6-/- mice

**Figure 3:**
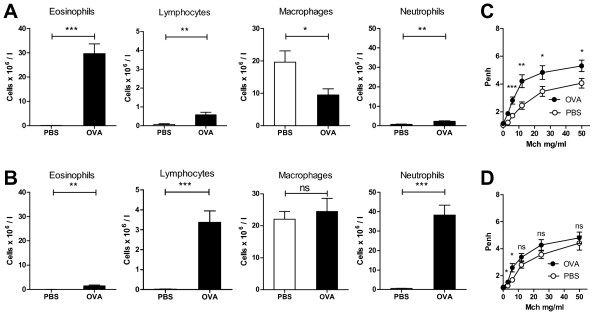
**BAL cell counts and the airway hyperreactivity of STAT4-/- and STAT6-/- mice**. A-B. Absolute numbers of eosinophils, lymphocytes, macrophages and neutrophils in BAL fluid in STAT4-/- (A) and STAT6-/- (B) mice. C-D. Bronchial hyperreactivity of STAT4-/- (C) and STAT6-/- (D) expressed as enhanced-pause values (Penh) for elevated concentrations of metacholine. RU = relative expression unit. Results are expressed as mean ± SEM. *p < 0.05, ** p < 0.01, *** p < 0.001. n = 8 mice/group, except n = 9 mice/STAT4-/- OVA group.

**Figure 4:**
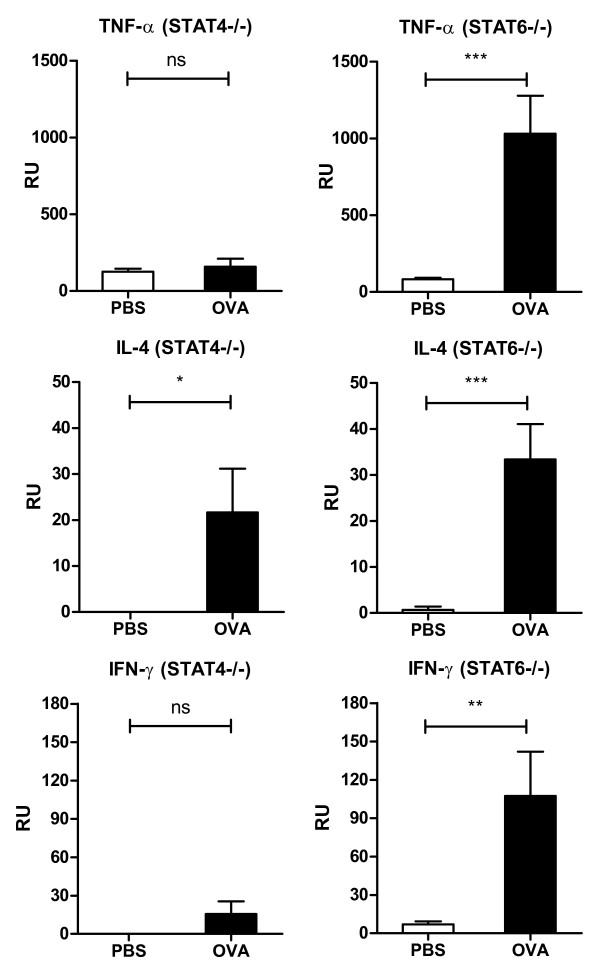
**Cytokine mRNA expression in the lungs of STAT4-/- and STAT6-/- mice**. The OVA-challenged STAT4-/- mice showed significantly elevated expression of IL-4 in the lung tissue, but the expression of TNF-α and IFN-γ was comparable to that of the controls. In the OVA-challenged STAT6-/- mice, the expression of TNF-α, IL-4 and IFN-γ was significantly higher than in the PBS-challenged mice. Results are expressed as mean ± SEM. RU = relative expression unit. *p < 0.05, **p < 0.01, *** p < 0.001. n = 8 mice/control group, n = 7 mice/study group.

The number of BAL eosinophils was significantly higher in the OVA-challenged STAT4-/- mice than in the PBS-challenged controls, with only a minor significant increase in the number of lymphocytes and neutrophils. There was also a minor significant decrease in the number of macrophages (Figure 3A). In contrast, an increase in the number of airway lumen eosinophils was minimal in the OVA-challenged STAT6-/- mice, but the influx of lymphocytes was comparable to that in the WT mice (Figure 3B). The number of neutrophils also was increased. Airway reactivity to methacholine was significantly stronger in the OVA-challenged STAT4-/- mice than in the PBS-challenged controls (Figure 3C). Compared to the PBS-challenged controls, the OVA-challenged STAT6-/- mice showed no airway hyperreactivity (Figure 3D). The expression of mRNA for major Th2 cytokine IL-4 was significantly elevated in the lung tissue of the OVA-challenged STAT4-/- mice, but we found no significant induction of proinflammatory cytokine TNF-α or Th1 cytokine IFN-γ (Figure 4). The mRNA expression of TNF-α, IFN-γ and IL-4, however, was significantly higher in the OVA-challenged STAT6-/- mice than in the PBS-challenged controls (Figure 4).

**Figure 5:**
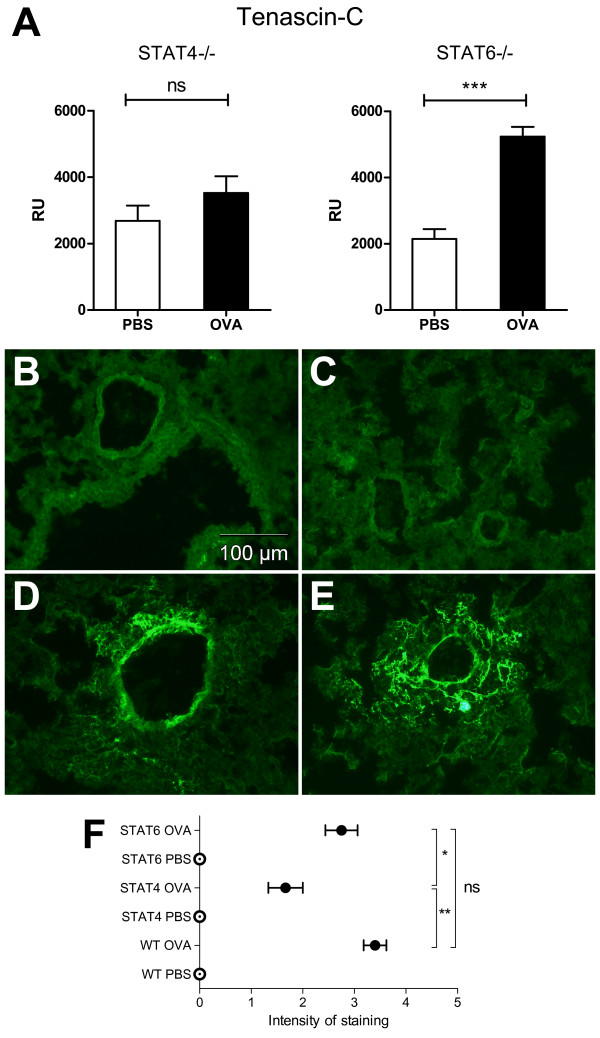
**mRNA expression and the immunoreactivity of tenascin-C in the lungs of STAT4-/- and STAT6 -/- mice**. (A) The relative mRNA expression of tenascin-C in the lungs of the control (n = 8 mice/group) and OVA-challenged (n = 7 mice/group) STAT4-/- and STAT6-/- mice. RU = relative expression unit. Results are expressed as mean ± SEM. B-E. Immunohistochemical staining of tenascin-C in frozen lung sections. PBS-challenged STAT4-/- (B) and STAT6-/- (C) mice had no tenascin-C immunoreactivity in the lung parenchyma. Lung sections from the OVA-challenged STAT4-/- mice (D) were only weakly tenascin-C immunoreactive, while OVA challenge strongly enhanced tenascin-C immunoreactivity in STAT6-/- mice (E). F. The semi-quantitative analysis of tenascin-C immunoreactivity in frozen sections. All the PBS-challenged controls were graded negative. OVA-challenged WT mice showed the most intensive expression of tenascin-C, although no significant difference was found in comparison with OVA-challenged STAT6-/- mice. In contrast, STAT4-/- mice exhibited a lower intensity of tenascin-C immunoreactivity than STAT6-/- and WT mice. 0 = no staining, 4 = very strong staining. n = 10 WT OVA; n = 9 WT PBS, STAT4-/- OVA; n = 8 STAT4-/- PBS, STAT6-/-PBS, STAT6-/- OVA. Circles represent the mean and the bars indicate the SEM. *p < 0.05, **p < 0.01, *** p < 0.001.

The expression of tenascin-C mRNA in the lung tissue of the OVA-challenged STAT4-/- mice remained unchanged and was comparable to that of the control mice (Figure 5A). In contrast, the expression of tenascin-C was significantly higher in the OVA-challenged than in the PBS-challenged STAT6-/- mice (Figure 5A). In lung sections, tenascin-C immunoreactivity was negative in the PBS-challenged STAT4-/- and STAT6-/- mice (Figure 5B and 5C respectively). The OVA-challenged STAT4-/- mice (Figure 5D) showed weaker immunoreactivity to tenascin-C than did their WT (Figure 2C) and STAT6-/- counterparts (Figure 5E). The localisation of immunoreactivity was quite similar in all the groups except that STAT4-/- and STAT6-/- mice had no OVA-induced tenascin-C expression in the veins as the WT mice did. Immunoreactivity to tenascin-C was strongly up-regulated in the alveolar structures and was found in small spots throughout the parenchyma of the OVA-challenged mice in the STAT6-/- group, corresponding to that of the WT mice. Semi-quantitative analysis of immunoreactivity for tenascin-C in the lung biopsies also showed diminished induction of tenascin-C expression by ovalbumin in STAT4-/- mice compared to WT and STAT6-/- mice (Figure 5F).

### TNF-α stimulation triggers enhanced levels of tenascin-C from the cultured human lung fibroblasts

**Figure 6:**
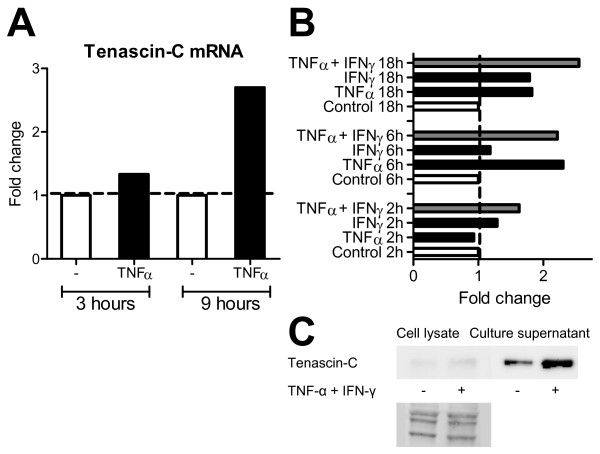
***In vitro *stimulation of human fibroblasts**. A. Recombinant TNF-α increases the mRNA expression of tenascin-C in foetal lung fibroblasts (MRC-9). Expression peaks at 9 hours after stimulation. The results are from one experiment. B. Recombinant TNF-α enhances the mRNA expression of tenascin-C also in human primary fibroblasts. TNF-α along with IFN-γ has a synergic effect, which further enhances the expression of tenascin-C mRNA. Control mRNA expressions of tenascin-C are adjusted to 1 and other expressions are shown as the fold change compared to the control. C. An immunoblot showing enhanced expression of protein for tenascin-C (M_r _290,000 band) in the primary fibroblasts (the first two lanes) and in the cell culture supernatant (the latter two lanes) after stimulation with TNF-α and IFN-γ for 18 h. Below SYPRO Ruby staining of the immunoblot confirming equal loading and transfer of proteins from cell lysate samples.

Our *in vivo *studies showed that tenascin-C expression was severely blunted in the airways of the OVA-challenged STAT4-/- mice. Moreover, the expression of major proinflammatory cytokine TNF-α was impaired in the lung tissue of the STAT4-/- mice. We therefore anticipated that impaired tenascin-C expression might stem from a lack of TNF-α stimulation in the airways of the OVA-challenged STAT4-/- mice. Indeed, the elevated expression of tenascin-C mRNA was evident in human lung fibroblasts (MRC-9) after stimulation with recombinant TNF-α (Figure 6A). This TNF-α-induced expression was time-dependent, peaking at 9 h.

In order to show that this increase in tenascin-C expression is not an MRC-9-specific phenomenon, we performed similar stimulations with human primary fibroblasts in which TNF-α also enhanced the expression of tenascin-C mRNA (Figure 6B). When the primary cells were cultured in the presence of TNF-α and with the major Th1 cytokine, IFN-γ, the expression of tenascin-C mRNA appeared to increase in a cumulative manner (Figure 6B). Further, the protein expression for tenascin-C was also elevated after the stimulation with TNF-α and IFN-γ for 18 h in the human primary fibroblasts as well as in the culture supernatant (Figure 6C).

## Discussion

Previous studies have demonstrated that prolonged allergic asthma may lead to structural changes in the airways, which include the modification of extracellular matrix proteins such as tenascin-C. In the present study we investigated the contribution of major Th1 and Th2 transcription factors (i.e. STAT4 and STAT6) to the regulation of expression profiles of tenascin-C in the asthmatic airways of mice. We found similar up-regulation of tenascin mRNA and protein in the OVA-challenged WT and STAT6-/- mice. Surprisingly, however, the OVA-challenged STAT4-/- mice showed no increase in tenascin-C mRNA production after allergen challenge, suggesting that STAT4 is an important regulator of tenascin-C in acute allergic asthma.

Tenascin-C is expressed both pre- and postnatally in the developing lung [5-7] and is thought to be involved in various inflammatory and fibrotic processes in the adult lung [3]. However, the exact mechanisms are still unknown. In the healthy adult human and mouse lung, tenascin-C is usually absent or its expression is sparse [5-8]. A coding single nucleotide polymorphism (SNP) in the tenascin-C gene has been linked to asthma in adulthood in the Japanese population [24]. In addition, cyclic strain, which is seen during bronchoconstriction, induces tenascin-C production through changes in the actin cytoskeleton [25]. Tenascin-C inhibits the contraction of the fibrin-fibronectin matrix implying a central role for tenascin-C in the modulation of myofibroblast contraction, which is important step in tissue repair [26]. The role of tenascin-C in asthma *in vivo *is largely unknown but aforementioned mechanisms shed some light on the possible role of tenascin-C in remodelling. In the present study, our OVA-challenged WT mice showed enhanced expression of tenascin-C in the alveolar structures and veins, whereas in the control mice, tenascin-C was almost completely absent. Accordingly, the expression of tenascin-C mRNA was higher in the lungs of the OVA-challenged mice than in the control mice. This is in accordance with the results of previous studies that have also shown diminished allergic inflammation in tenascin-C-negative mice [27].

In human adult bronchi, tenascin-C expression is markedly higher in asthma patients in a stable phase and even immediately after specific antigen challenge [8,28,29]. The accumulation of tenascin-C is associated with atopic but not with nonatopic asthma [9,30], and the thickness of the tenascin-C layer correlates with inflammatory cells in the lamina propria in atopic asthma [9]. In the ovalbumin-induced mouse allergy model and in patients with atopic asthma, the Th2 type of inflammation prevails in the airways. Th1 cells, IFN-γ and TNF-α are considered important in severe asthma [1,31]. No published studies have shown association between asthma severity and tenascin-C expression to our knowledge. To test whether Th1 or Th2 cells are important regulators of tenascin-C expression in experimental allergic asthma, we studied the responses of mice with impaired Th1 (STAT4-/-) or Th2 (STAT6-/-) immunity. To our surprise, our results revealed that tenascin-C expression was normal in STAT6-deficient mice but severely impaired in STAT4-deficient mice, thus suggesting that STAT4 plays a critical role in tenascin-C regulation. IFN-γ and TNF-α are known stimulators of tenascin-C expression in bronchial epithelial cells [18], and at least TNF-α in fibroblasts [19]. The low expression of tenascin-C in our STAT4-/- mice may therefore be related to the lack of IFN-γ and TNF-α in these mice. This is also in line with our *in vitro *results, which demonstrated that the stimulation of cultured human MRC-9 fibroblasts with recombinant TNF-α or with TNF-α and IFN-γ together from human primary fibroblasts induced the expression of tenascin-C. Thus, the present results suggest that proinflammatory cytokine TNF-α and Th1 cytokine IFN-γ released into the airways after allergen challenge critically regulate tenascin-C expression and may therefore play an important role in initiating the remodelling process of the airways. Our results, together with previous studies that have shown diminished expression of Th2 cytokines in tenascin-C-negative mice [27], also support the role of tenascin-C as possible mediator of inflammation.

## Conclusions

We conclude that the first steps in the remodelling of the airways are already evident in this mouse acute allergic asthma model. After four weeks of challenge, we found up-regulation of tenascin-C in the WT mice. The STAT4-/- mice exhibited lower mRNA expression for tenascin-C, TNF-α and IFN-γ than did the WT mice. This reinforces the role of STAT4 in allergic inflammation in addition to the rather well characterised role of STAT6. The present results are consistent with those of previous reports suggesting that the blocking of STAT6 may make it possible to inhibit allergen-induced AHR responses and pulmonary eosinophilia. However, our results also show that the expression of tenascin-C, a possible early initiator of remodelling, is instead regulated by the STAT4 transcription factor and TNF-α with Th1 cytokine IFN-γ in allergic airway inflammation. Moreover, this study, as well as previous ones [32,33] raises the question of the effectiveness of blocking only the STAT6 pathway as a treatment for asthma. The role of tenascin-C in the diagnosis and treatment of inflammatory airway diseases remains to be explored, but tenascin-C may be a candidate for an early marker of the remodelling process.

## Competing interests

The authors declare that they have no competing interests.

## Authors' contributions

AM participated in handling the knockout mice, carried out the RNA studies on the knockout mice, studied the tissue samples from all the mice, participated in analysing the data and designing the study and drafted the article. PK participated in designing the study, handling the knockout mice and drafting the article. ML performed the RNA work, and handled the WT mice and participated in drafting the article. TS performed the human *in vitro *studies and drafted the article. KS participated in the *in vitro *studies. M-LM participated in the RNA studies and drafted the article. PP participated in drafting the article. IV participated in drafting the article and helped with analyzing the tissue samples. MM participated in drafting the article. AL participated in designing the study and drafting the article. LAL participated in drafting the article, and HA carried out the design of the study, participated in analyzing the data and drafted the article. All authors read and approved the final manuscript.
